# Supplementary material for: Data Mining for Three-Dimensional Organic Dirac Materials: Focus on Space Group 19
Source: Sci Rep. 2017 Aug 4;7:7298. doi: 10.1038/s41598-017-07374-7 (PMC5544778; doi:10.1038/s41598-017-07374-7)
Supplement: Supplementary file 1 — Supplementary Material [file 41598_2017_7374_MOESM1_ESM.pdf]

# Supplementary Material: Data Mining for Three-Dimensional Organic Dirac Materials: Focus on Space Group 19

**R. Matthias Geilhufe[1,*], Stanislav S. Borysov[1], Adrien Bouhon[2], and Alexander V. Balatsky[1,3,4]**

[*]geilhufe@kth.se
[1]Nordita, Center for Quantum Materials, KTH Royal Institute of Technology and Stockholm University, Roslagstullsbacken 23, SE-106 91 Stockholm, Sweden
[2]Department of Physics and Astronomy, Uppsala University, Box 516, SE-751 20 Uppsala, Sweden
[3]Institute for Materials Science, Los Alamos National Laboratory, Los Alamos, NM 87545, USA
[4]ETH Institute for Theoretical Studies, ETH Zürich, 8092 Zürich, Switzerland

In the following, the band structures and molecular structures of the mined materials are presented which were not discussed in the main manuscript. The materials $C_{10}H_{10}Br_2Cl_3NO_2$[1], $C_{12}H_{13}NO_2$[2], and $C_{13}H_{12}N_2O$[3] contain the discussed Dirac crossings in the valence band close to the highest occupied electronic state and are plotted in Figure 2. The distance of the crossing to the Fermi-level is in the range of 100-200 meV. Within the materials $C_9H_{10}F_3NO$[4] and $C_{10}H_{12}BrNO$ [5], similar crossings can be

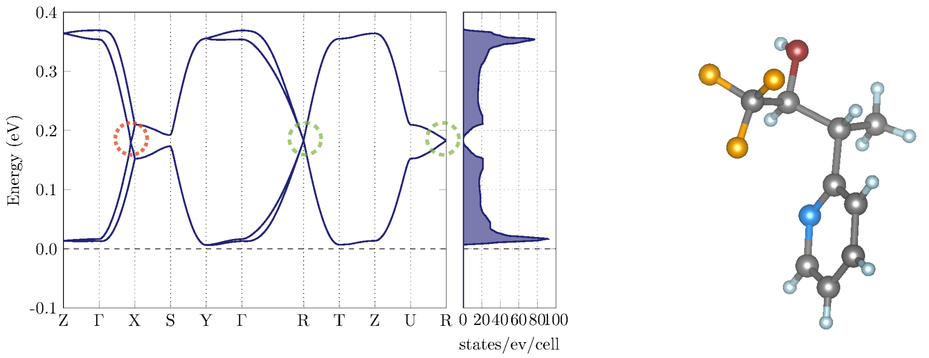


**(a)** $C_9H_{10}F_3NO$

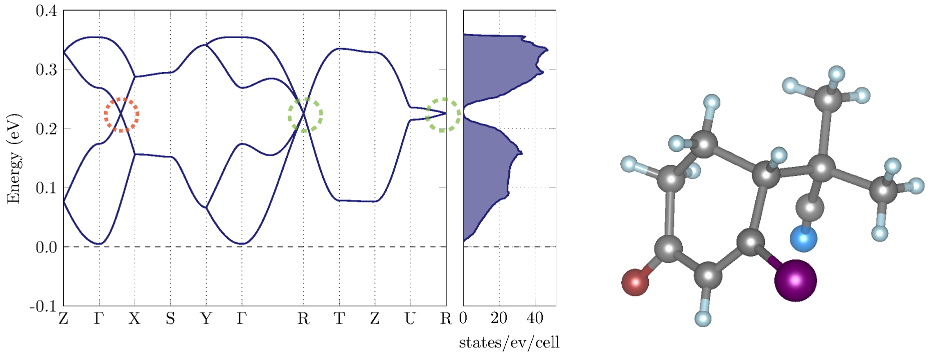


**(b)** $C_{10}H_{12}BrNO$

**Figure 1. Electronic structure and molecular structure of materials with Dirac crossings in the conduction band.** The chemical elements are indicated by the colors: carbon (gray), hydrogen (light-blue), nitrogen (blue), oxygen (red), fluorine (yellow) and bromine (purple).

found in the conduction band. The distance of the crossing to the lowest unoccupied state is about 200 meV, as can be seen in Figure 1.

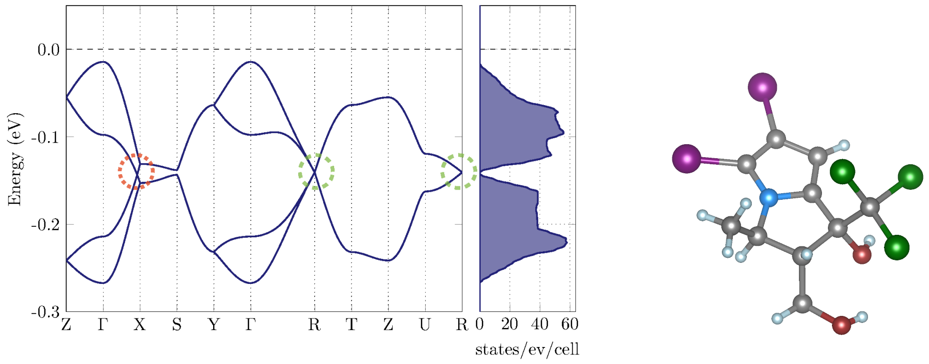


**(a)** $C_{10}H_{10}Br_2Cl_3NO_2$

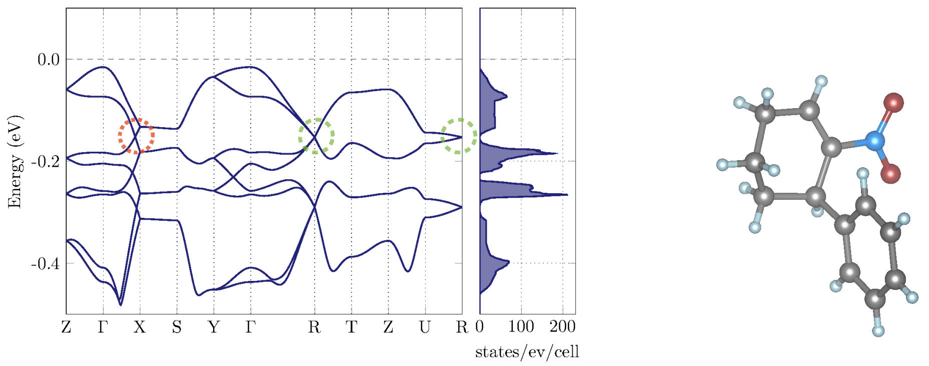


**(b)** $C_{12}H_{13}NO_2$

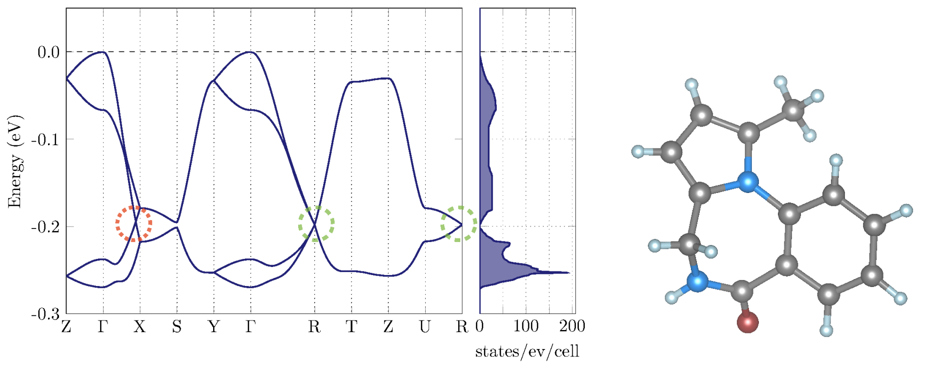


**(c)** $C_{13}H_{12}N_2O$

**Figure 2. Electronic structure and molecular structure of materials with Dirac crossings in the valence band.** The colors indicate: gray: carbon, light-blue: hydrogen, blue: nitrogen, red: oxygen, purple: bromine, green: chlorine.

## References


1. Bae, J.-Y., Lee, H.-J., Youn, S.-H., Kwon, S.-H. & Cho, C.-W. Organocatalytic asymmetric synthesis of chiral pyrrolizines by cascade conjugate addition- aldol reactions. *Org. letters* **12**, 4352–4355 (2010).
2. Dong, L. *et al.* Asymmetric nitroallylation of arylboronic acids with nitroallyl acetates catalyzed by chiral rhodium complexes and its application in a concise total synthesis of optically pure (+)-$\gamma$-lycorane. *Org. letters* **7**, 4285–4288 (2005).
3. Butin, A. V. *et al.* Furan ring opening–pyrrole ring closure: a new synthetic route to aryl (heteroaryl)-annulated pyrrolo [1, 2-a][1, 4] diazepines. *Org. & biomolecular chemistry* **8**, 3316–3327 (2010).

**4.** Šterk, D., Stephan, M. & Mohar, B. Highly enantioselective transfer hydrogenation of fluoroalkyl ketones. *Org. letters* **8**, 5935–5938 (2006).

**5.** Eloi, A. *et al.* Cationic planar chiral ($\eta$6-arene) mn (co) 3+ complexes: resolution, nmr study in chiral-oriented solvents, and applications to the enantioselective synthesis of 4-substituted cyclohexenones and ($\eta$6-phosphinoarene) mn (co) 3+ complexes. *Organometallics* **29**, 3876–3886 (2010).
